# Examining the Development of Information Needs Assessment Questionnaires in Oncology: Protocol for a Scoping Review

**DOI:** 10.2196/35639

**Published:** 2022-09-01

**Authors:** Maclean Thiessen, Daranne Harris, Patricia Tang, Shelley Raffin Bouchal, Shane Sinclair

**Affiliations:** 1 Department of Internal Medicine Rady Faculty of Health Sciences University of Manitoba Winnipeg, MB Canada; 2 Faculty of Nursing University of Calgary Calgary, AB Canada; 3 Cumming School of Medicine University of Calgary Calgary, AB Canada

**Keywords:** information needs, cancer, patient-oriented research, psychometric, measure, questionnaire, oncology

## Abstract

**Background:**

Information needs are one of the most prevalent unmet supportive care needs of those living with cancer, including patients and their informal caregivers. Understanding how existing questionnaires for evaluating information needs have been developed is important for guiding appropriate use and informing future research. A literature review examining how information needs assessment questionnaires for use in the cancer context have been developed, with a specific focus on how questionnaire items have been identified, does not exist.

**Objective:**

This scoping review will examine how questionnaires for assessing the information needs of those living with cancer have been developed with special focus on how patients, informal caregivers, and health care professionals have been involved in the selection and identification of questionnaire items.

**Methods:**

This review will include published studies describing the development and validation of information needs assessment questionnaires for use in the oncology context. MEDLINE (Ovid), Embase (Ovid), CINAHL, Scopus, Web of Science, the Cochrane Database of Systematic Reviews, and PsycInfo will be searched. Articles published at any point up to the date of the search will be eligible for inclusion. One person will screen titles and abstracts, and 2 people will screen and extract data from full-text articles.

**Results:**

Results are expected to be available in early 2023. Summary tables and a narrative summary will be used to describe results.

**Conclusions:**

This scoping review will assist in identifying appropriate information needs assessment tools to incorporate into clinical and research contexts in oncology. It will also identify if additional information needs assessment tools are needed.

**International Registered Report Identifier (IRRID):**

PRR1-10.2196/35639

## Introduction

### Overview

Information needs are one of the most commonly unmet supportive care needs of patients and informal caregivers (ie, friends and family who provide unpaid support to patients) [1]. Information plays an important role in both emotional and problem-based coping [2]. When faced with a health problem such as a new diagnosis of cancer, individuals seek information to help them adjust and understand what actions they can take to improve their situation both in the short and long term. When information needs are addressed, patients are more likely to be active participants in decision-making [3], have better health-related quality of life, and lower rates of anxiety and depression [4,5].

Information needs of patients with cancer and their informal caregivers have been assessed in multiple studies, using validated questionnaires [6,7]. Validated questionnaires provide researchers with tools that have been rigorously developed [8] and produce data that can be compared between populations and across time. However, when assessing information needs using a validated questionnaire, it is important to understand how the questionnaire was developed and its intended use. Inappropriate selection of questionnaires can lead to erroneous conclusions and recommendations [8].

One important consideration when selecting a questionnaire is how the questionnaire items were identified. Regarding information needs, at least on a theoretical level, an important distinction is between normative and expressed information needs [9]. The word “normative” [2] has been used to describe the information needs identified by health care professionals as important for health care recipients to know. In contrast, “expressed” [2] needs refer to information needs that are identified as important by health care recipients such as patients with cancer and their informal caregivers. Although there is likely an overlap between normative and expressed needs, it is hypothesized that key differences are also likely to exist both in the content of these different types of information needs and the consequences of whether or not each type of information need is met.

Normative information needs may, at least to some degree, be influenced by the pressures that health care professionals face in their respective clinical, research, and administrative roles. On the other hand, expressed information needs may be more likely to reflect day-to-day challenges of those living with cancer, as they continue to pursue their prediagnosis value-based goals [9-11], while navigating a cancer diagnosis, survivorship, and a health care system with its own goals and values. The crux of the distinction is that, at least theoretically [9], an educational intervention designed to address normative information needs may facilitate a patient fitting well within the health care system, whereas targeting expressed needs may facilitate health care fitting better with the patient’s life and values. If this is true, when selecting or interpreting the results from a questionnaire designed to assess information needs, it is important to have a clear understanding of whether the questionnaire is assessing normative or expressed information needs. This likely requires an understanding of the intended use of the questionnaire, the steps involved in its development, how it was validated, and the processes involved in identifying the questionnaire items, including how health care recipients, informal caregivers, and health care professionals were involved in questionnaire item generation and selection.

A preliminary search of MEDLINE, the Cochrane Database of Systematic Reviews, *JBI Evidence Synthesis,* and PROSPERO was conducted to identify previous systematically conducted literature reviews exploring how information needs assessment tools in oncology were developed. This search identified systematic reviews of information needs in the cancer population that included studies using validated information needs assessment tools to describe the information needs of patients with cancer [6,7]. Additionally, one review by Christalle et al [12] was identified that had systematically reviewed information needs assessment tools across the health care spectrum, including in the cancer context. However, similar to other reviews of health needs assessment tools [13,14], this review focused on the methodological quality and psychometric properties of the tools. A review specifically exploring how questionnaire items were identified and selected and who was involved in this process could not be identified. Therefore, a review is needed that is specific to the cancer context, characterizing how information needs assessment questionnaires have been developed, their intended use, and whether the types of information needs being assessed are likely normative, expressed, or both. The preliminary review performed as part of the development of this protocol supports that there are adequate numbers of information needs assessment questionnaires used in contemporary cancer research to provide data for this review as evidenced by the 11 oncology-specific questionnaires identified in the review by Christalle et al [12].

This review will use a scoping review approach. A scoping review is the most appropriate method for examining how information needs assessment tools in the oncology context have been developed. Scoping reviews are a rigorous approach to knowledge synthesis and are also flexible and can be used to address a number of different types of objectives, including mapping the literature and describing how research has been conducted [15,16]. This contrasts with systematic reviews, which are best suited for research questions related to clinical practice, where a comprehensive and unbiased summary of the literature is required [15,17,18], such as when results from randomized controlled trials are being compared to determine best practices.

The objective of this scoping review is to examine how the existing tools for assessing information needs of patients with cancer and their friends and family have been developed, including how they have incorporated expressed information needs. This will be achieved by systematically reviewing the literature to comprehensively identify information needs assessment tools developed for the cancer context and then examining how they have been developed and validated. The rationale for the development of each questionnaire as well as the processes for identifying, finalizing, and validating the questionnaire will be described. Regarding expressed information needs, the role of the patients and informal caregivers in identifying potential questions and needs domains as well as determining the final version of the assessment tool will be summarized.

### Review Questions

The objectives of this review will be achieved by systematically reviewing the literature to answer the following questions:

- What questionnaires have been created and validated for evaluating the information needs of people living with cancer?
  - What is the stated purpose of each questionnaire?
  - What cancer contexts (ie, cancer type, treatment intent, and population) have these tools been developed for?
- How were the questionnaires developed?
  - How were potential questionnaire items identified and finalized?
  - How were the questionnaires validated?
  - How were patients, health care professionals, and informal caregivers involved in the process of developing the questionnaires, including in the identification and selection of questionnaire items?
  - How were test and measurement guidelines (eg, COSMIN checklist [19]) used in the development and reporting of the measure?

## Methods

The proposed scoping review will be conducted in accordance with the JBI methodology for scoping reviews [16,20]. The one exception to this is that the screening of titles and abstracts will be conducted by a single author, as supported by Cochrane [21].

### Eligibility Criteria

#### Population

This scoping review is focused on characterizing the development of validated assessment tools rather than characterizing differences in measured outcomes in certain populations. As such, the “participants” aspect of the scoping review eligibility criteria is not applicable.

#### Concept

This scoping review will examine how information needs assessment tools have been developed, including the motivation for the development, the steps in the development, and the steps taken to include the expressed information needs of health care recipients.

#### Context

This scoping review will include the literature relevant to the cancer context, both in clinical and research settings. It will include published reports describing the development of tools designed for patients and/or informal caregivers (ie, friends and family). Literature specific to the pediatric population will be excluded. Non–English-language studies will be excluded.

#### Types of Sources

This scoping review will consider any report related to the development of information needs questionnaires for patients with cancer published in peer-reviewed journals. Reports will include those that directly describe and report on their development, including methods of identifying questionnaire items as well as testing of psychometric properties. Additionally, reports cited as rational for selection of certain items will be included. As a result, this review will include a wide range of reports including but not limited to the following: protocols of both experimental and quasi-experimental study designs such as randomized controlled trials, nonrandomized controlled trials, before and after studies, and interrupted time series studies; analytical observational studies including prospective and retrospective cohort studies, case-control studies, and analytical cross-sectional studies; descriptive observational study designs including case series, individual case reports, and descriptive cross-sectional studies; experimental studies; reports on preliminary results and works in progress; qualitative studies; systematic reviews; and peer-reviewed essays and opinion papers.

#### Search Strategy

The search strategy will aim to locate both published and unpublished studies related to the development of information needs assessment tools for the oncology context. An initial limited search of MEDLINE (Ovid) and CINAHL Plus with Full Text was undertaken to identify articles on the topic. The text words contained in the titles and abstracts of relevant articles and the index terms used to describe the articles were used, in collaboration with a health sciences librarian, to develop a full search strategy for MEDLINE (Ovid) and CINAHL (Multimedia Appendix 1). The search strategy, including all identified keywords and index terms, will be adapted for each included database or information source. The databases to be searched include MEDLINE (Ovid), Embase (Ovid), CINAHL, Scopus, Web of Science, the Cochrane Database of Systematic Reviews, and PsycInfo.

Studies published in English will be included. Non–English-language studies will not be included as the researchers are primarily interested in learning what tools are available for use in their respective English-based clinical and research practices. Studies published since the beginning of the database will be included, as there is no reason to exclude older studies.

As appropriate, authors of reports will be contacted to determine if missing or additional data are available in peer-reviewed publications. Grey literature, and non–peer-reviewed reports, including unpublished studies or protocols, will not be excluded from this review. Inclusion and exclusion criteria are summarized in Textbox 1.

**Inclusion and exclusion criteria for search strategy.**

**Inclusion criteria**

- Reports indexed up to the date when article searching begins (ie, post completion of blind protocol peer review).
- Reports describing the development or use of information needs assessment questionnaires, specifically for adults living with cancer, including patients and informal caregivers.
- Reports related to any type of malignancy, including a single or multiple types.
- Reports related to any point in the cancer journey, from diagnosis to surveillance or palliation.
- Any geographic location.

**Exclusion criteria**

- Non–peer-reviewed literature.
- Non–English-language literature.
- Reports related to the development of multidimensional needs assessment tools (ie, not focused on information needs).
- Reports related to tools designed specifically for the pediatric population, including adult informal caregivers of patients with pediatric cancer.
- Reports related to assessing information needs regarding cancer screening.

### Evidence Selection

Following the initial database search, all identified citations will be collated and uploaded into Covidence (Veritas Health Innovation), and duplicates will be removed. Titles and abstracts will then be screened by 1 independent reviewer for assessment against the inclusion criteria for the review [21]. The full text from the screened articles will be assessed in detail against the inclusion criteria by 2 independent reviewers. Reasons for the exclusion of full-text sources of evidence that do not meet the inclusion criteria will be recorded and reported in the scoping review. Any disagreements that arise between the reviewers at each stage of the selection process will be resolved through discussion or with an additional reviewer(s). The results of the search and the study inclusion process will be reported in full in the final scoping review and presented in a PRISMA-ScR (Preferred Reporting Items for Systematic Reviews and Meta-analyses extension for Scoping Reviews) flow diagram [22]*.*

Data will be extracted from papers identified through the search strategy by 2 independent reviewers, using a data extraction tool developed by the reviewers. The data extracted will include specific details about the participants, concept, context, study methods, and key findings relevant to the review questions.

A draft extraction form is provided (Multimedia Appendix 2)*.* It was initially developed from the template provided by JBI for data extraction tools used in scoping reviews [16] and informed by the research questions. In particular, specific data extraction questions focused on identifying the level of involvement of patients and informal caregivers, compared to health care professionals, will assist in evaluating whether the questionnaire is focused on assessing expressed or normative information or a balance of both, or if it is simply not clear from available literature. Additionally, the COSMIN checklist sections related to general recommendations and content validity were used to inform the development of the data extraction tool [19], as they closely relate to the objectives of this study.

The draft data extraction tool will not be piloted prior to data extraction. However, the extraction tool is expected to be modified and revised during the process of extracting data to capture relevant data, including data that emerges as important during the course of data extraction. Modifications to the extraction tool will be detailed in the scoping review. Any disagreements that arise between the reviewers will be resolved through discussion or via additional independent reviewers.

Of note, to ensure that the number of information needs assessment tools reviewed in this study is as comprehensive as possible, the titles and abstracts identified through the initial database search will also be reviewed to identify studies reporting on quantitative assessments of information needs using validated questionnaires. Screening for these articles will be accomplished by a single reviewer who will also review the full text of these studies, including their references lists, to identify additional reports potentially meeting the inclusion criteria of this scoping review. These articles will be combined with other articles selected for full-text review to meet inclusion criteria, and from that point, they will be treated equally with articles identified directly through the database search. The number of articles identified through this process will be clearly demarcated in the PRISMA-ScR flow diagram.

## Results

Activities related to this scoping review began in December 2021 with the drafting and submission of this protocol for peer review and publication. Results are expected to be available in early 2023 and will be reported in accordance with the PRISMA-ScR reporting guidelines [22]. Extracted data will be presented in both narrative and table forms. A summary table of the year of publication, country of the lead author, and cancer contexts (ie, treatment intent, type of cancer, and during active treatment or surveillance) for which the questionnaires were developed will be created. Additionally, 2 separate tables will be created summarizing the collected data related to the first and second research questions.

## Discussion

### Principal Findings

Based on the preliminary search conducted as part of the development of this protocol, the resulting scoping review will be the first to systematically evaluate the development of information needs assessment questionnaires for use in the oncology context. Importantly, it will characterize how the expressed needs of those living with cancer have been incorporated into the existing information needs assessment tools. As such, this review has the potential to impact both clinical and research practices in oncology, including but not limited to the development of more rigorous patient-reported measures in oncology settings.

In the clinical setting, this review will be helpful in guiding tool selection for capturing information needs in routine practices. Screening for psychosocial distress as part of the routine oncology clinical practice is considered standard of care by many professional organizations such as the American Society of Clinical Oncology [23]. Routinely, patient-reported outcome measures (PROMs) are central to distress screening strategies. In some institutions, PROMs that specifically assess information needs are collected as part of a routine practice [24]. By being the first systematically conducted review to characterize whether existing information needs assessment tools developed for the cancer context assess normative versus expressed information needs, this review will inform clinicians in identifying which information needs questionnaires to include as part of their routine assessments. Additionally, it will assist clinicians in the correct interpretation of results, which may lead to better identification of information gaps and development of improved information provision practices.

From a research perspective, this review is expected to support researchers in identifying appropriate tools for capturing information needs–related data and facilitating awareness of the limitations of the selected tools [8]. It will also identify where there is a need for development of additional measures and provides insight into best practices for the development of information needs measures in the future. Lastly, by identifying how the expressed information needs [2] of those experiencing cancer have been included in existing measures, this review will provide an important lens for interpreting the existing published literature characterizing the information needs of those living with cancer.

### Limitations

Despite identifying what appears to be an adequate body of literature to support this review, it is not clear whether sufficient details will be able to be identified in the existing peer-reviewed literature to adequately address the research questions. Although the rate of publication of protocols is increasing [25], research results, including descriptions of the research methods employed, commonly go unpublished [26]. It is simply not known whether a sufficient level of detail about the procedures used to develop the instruments to answer the research questions will be identified in the peer-reviewed literature. Identifying the relative presence or absence of the details relevant to the research questions in the literature is not an explicit objective of this review; however, the discovery of insufficient data to address specific research questions will certainly be important for guiding future work such as in-depth qualitative explorations of how existing questionnaires have been developed incorporating semistructured interviews with the lead developers.

### Conclusions

Information needs are one of the most commonly unmet supportive care needs of those living with cancer [1]. Unmet information needs negatively impact the cancer experience [3-5]. Understanding how the questionnaires used to assess the information needs of those living with cancer have been developed is key to appropriate questionnaire selection and interpretation of reported results [8]. Systematic literature reviews exploring various aspects of information needs questionnaires exist [13,14], and they have included tools specific to oncology [12]; however, a review is needed to specifically explore how information needs assessment questionnaires in the oncology context have been developed. This review will address this gap in the literature, and in doing so, assist future work to better support those living with cancer.

## Appendix

Multimedia Appendix 1 Sample search strategies.

Multimedia Appendix 2 Data extraction tool.

## Abbreviations

PRISMA-ScR: Preferred Reporting Items for Systematic Reviews and Meta-analyses extension for Scoping Reviews
PROM: patient-reported outcome measure
