# Supplementary material for: Examining the Development of Information Needs Assessment Questionnaires in Oncology: Protocol for a Scoping Review
Source: JMIR Res Protoc. 2022 Sep 1;11(9):e35639. doi: 10.2196/35639 (PMC9478820; doi:10.2196/35639)
Supplement: Multimedia Appendix 1 [file resprot_v11i9e35639_app1.docx]

## Appendix I: Search strategy

| Ovid MEDLINE(R) and Epub Ahead of Print, In-Process, In-Data-Review & Other Non-Indexed Citations and Daily <1946 to November 24, 2021> | | |
| --- | --- | --- |
| Search Date: November 26, 2011 | | |
| 1 | (information* or education*).mp. [mp=title, abstract, original title, name of substance word, subject heading word, floating sub-heading word, keyword heading word, organism supplementary concept word, protocol supplementary concept word, rare disease supplementary concept word, unique identifier, synonyms] | 2393938 |
| 2 | (assessment scale or questionnaire or survey or instrument).mp. [mp=title, abstract, original title, name of substance word, subject heading word, floating sub-heading word, keyword heading word, organism supplementary concept word, protocol supplementary concept word, rare disease supplementary concept word, unique identifier, synonyms] | 1068725 |
| 3 | (validation or validity or development or reliability).mp. [mp=title, abstract, original title, name of substance word, subject heading word, floating sub-heading word, keyword heading word, organism supplementary concept word, protocol supplementary concept word, rare disease supplementary concept word, unique identifier, synonyms] | 3451006 |
| 4 | (cancer* or oncology* of hodgkin* or neoplas* or lymphoma or leukemia).mp. [mp=title, abstract, original title, name of substance word, subject heading word, floating sub-heading word, keyword heading word, organism supplementary concept word, protocol supplementary concept word, rare disease supplementary concept word, unique identifier, synonyms] | 3981125 |
| 5 | 1 and 2 and 3 and 4 | 3487 |
| 6 | exp Neoplasms/ | 3576090 |
| 7 | exp Patient Education as Topic/ | 87931 |
| 8 | exp Data Collection/ | 2379094 |
| 9 | exp *"Reproducibility of Results"/ or exp *"Surveys and Questionnaires"/ or exp *Psychometrics/ | 243139 |
| 10 | 6 and 7 and 8 and 9 | 661 |
| 11 | 5 or 10 | 4114 |
| 12 | limit 11 to (English and journal article and medline) | 3434 |
| Limiters: English, Journal Article, MEDLINE | |  |

| CINAHL Plus with Full Text | | |
| --- | --- | --- |
| Search Date: Friday, November 26, 2021 | | |
| S1 | (TI ( cancer* or oncology* ) OR AB ( cancer* or oncology* )) OR (TI *hodgkin* OR AB *hodgkin*) OR (TI neoplas* OR AB neoplas*) OR (TI lymphoma* OR AB lymphoma*) OR (TI leukemia* OR AB leukemia*) | 518,998 |
| S2 | (TI information* N2 need* OR AB information* N2 need*) OR (TI information* OR AB information*) OR (TI patient education OR AB patient education) | 492,439 |
| S3 | (TI assessment scale OR AB assessment scale) OR (TI ( questionnaire or survey or scale or instrument ) OR AB ( questionnaire or survey or scale or instrument )) | 697,067 |
| S4 | (TI ( validation or validity ) AND AB ( validation or validity ) ) OR (TI development AND AB development ) OR (TI reliability AND AB reliability ) | 69,713 |
| S5 | S1 AND S2 AND S3 AND S4 | 282 |
| S6 | (MM "Neoplasms+") | 527,065 |
| S7 | (MM "Information Needs") OR (MM "Needs Assessment") OR (MM "Patient Education+") | 4,583 |
| S8 | (MM "Structured Questionnaires") OR (MH "Surveys+") OR (MM "Instrument Construction+") OR (MM "Instrument Validation") OR ((MM "Measurement Issues and Assessments+") OR (MM "Psychometrics") ) | 259,356 |
| S9 | S6 AND S7 AND S8 | 66 |
| S10 | S5 AND S9 | 346 |
|  |  |  |
| Limiters: | English, Scholarly (Peer Reviewed Journals) |  |
