# Supplementary material for: Examining the Development of Information Needs Assessment Questionnaires in Oncology: Protocol for a Scoping Review
Source: JMIR Res Protoc. 2022 Sep 1;11(9):e35639. doi: 10.2196/35639 (PMC9478820; doi:10.2196/35639)
Supplement: Multimedia Appendix 2 [file resprot_v11i9e35639_app2.docx]

Appendix II: Data extraction instrument

**Scoping Review Details**

**Scoping Review Title:** Examining the development of information needs assessment questionnaires in oncology: A scoping review protocol

**Review Objective/s:** Examine how questionnaires for assessing the information needs of those living with cancer been developed.

**Review Question/s:**

1. What questionnaires have been created and validated for evaluating the information needs of people living with cancer?

1. What is the stated purpose of each questionnaire?

b. What cancer contexts (i.e., cancer type, treatment intent, population) have these tools been developed for?

1. How were the questionnaires developed?

a. How were potential questionnaire items identified and finalized?

b. How were the questionnaires validated?

c. How were patients, healthcare professionals, and informal caregivers involved in the process of developing the questionnaires, including in the identification and selection of questionnaire items?

d. How were test and measurement guidelines, such as the COSMIN Checklist, used in the development and reporting of the measure?

**Population/Concept/Context Framework**

**Population** – n/a

**Concept** - examine how information needs assessment tools have been developed, including the motivation for the development, stages of development, and the process taken to include the expressed information needs of healthcare recipients.

**Context** - the literature relevant to the cancer context, both in clinical and research settings. It will include published reports describing the development of tools designed for patients, informal caregivers (i.e., friends and family), or both. Literature specific to the paediatric population will be excluded. Non-English language studies will be excluded.

**Inclusion criteria**

Reports indexed up to date when article searching begins (i.e., post completion of blind protocol peer review).

Reports describing the development OR use of information needs assessment questionnaires specifically for adults living with cancer, including patients and/or informal caregivers.

Reports related to any type of malignancy, including a single or multiple types.

Reports related to any point in the cancer journey, from diagnosis to surveillance or palliation.

Any geographic location.

**Exclusion criteria**

Non-peer reviewed literature.

Non-English literature.

Reports related to the development of multi-dimensional needs assessment tools (i.e., not focused on information needs).

Reports related to tools designed specifically for the pediatric population, including for adult informal caregivers of pediatric cancer patients.

Reports related to assessing information needs regarding cancer screening.

**Details/Results extracted from source of evidence**

(in relation to the concept of the scoping review)

Citation Details (author/s, date, title, journal, volume, issue, pages)

Country

Context

Participants

Aim of manuscript

Questionnaire Name

Questionnaire Aim

Intended Context

How are information/information needs described/defined?

Was there a guiding theory/conceptual framework?

What was the process for developing the questionnaire?

How were the patients/informal caregivers/healthcare professionals identified for participation in questionnaire development?

How were patients/informal caregivers/healthcare providers involved in questionnaire item identification/selection? Did the questionnaire items come from a different source?

| Based on the process used to identify information needs questionnaire items, place an x in the category that best describes the nature of the information needs assessed: | | | | | | |
| --- | --- | --- | --- | --- | --- | --- |
| Questionnaire items reflect: | Normative information needs (i.e., questionnaire items identified and selected by healthcare professionals) | Mostly normative information needs (i.e., some/minimal input from patients/informal caregivers, most influence of item selection from healthcare professionals) | Balanced mix of normative/expressed information needs (i.e., patients/informal caregivers and healthcare providers partnered or were provided relatively equal opportunity for influence on the selection of items) | Mostly expressed information needs (i.e., some/minimal input from healthcare providers, most influence of item selection from patients/informal caregivers) | Expressed information needs (i.e., questionnaire items identified and selected by patients/informal caregivers) | Unable to tell the relative level of influence healthcare providers and patients/informal caregivers had on item identification and selection |
| X most appropriate: |  |  |  |  |  |  |

What were the demographics of the individuals who developed the questionnaire or participated in developing the questionnaire?

What was reported regarding test/measurement guidelines, including testing for structural validity, internal consistency, cross-cultural validity/measurement invariance, measurement error and reliability, criterion validity, hypothesis testing for construct validity, responsiveness, and translation process?
